# Supplementary material for: Radical Scavenging-Linked Anti-Adipogenic Activity of Aster scaber Ethanolic Extract and Its Bioactive Compound
Source: Antioxidants (Basel). 2020 Dec 16;9(12):1290. doi: 10.3390/antiox9121290 (PMC7766398; doi:10.3390/antiox9121290)
Supplement: Supplementary file 1 [file antioxidants-09-01290-s001.pdf]

Table S1. HPLC-Q-Tof conditions for ASE analysis

**Instrument**	**Conditions**		
Column	CAPCELL PAK C18 UG120 S5<br>(4.6 mm x 250 mm x 5.0 μm, OSAKA SODA, Japan)		
Column temp.	28℃		
Mobile phase (Gradient)	Time (min)	A<br>1% acetic acid in water<br>(%)	B<br>Acetonitrile<br>(%)
	0	90	10
	28	60	40
	39	40	60
	40	90	10
	45	90	10
Detector	Diode Array Detector (DAD 3000) (272 nm)		
Flow rate	0.7 mL/min		
Run time	55 min		
Injection volume	20 μL		

Table S2. Q-TOF condition (Agilent 6550 Q-TOF with agilent 1260 HPLC)

Instrument	Conditions
Drying gas (N_2) flow rate	17 L/min
Drying gas (N_2) temperature	225 ℃
Nebulizer pressure	45 psi
Sheath gas temperature	350 ℃
Sheath gas flow	11 L/min
Capillary voltage	3,500 V
Nozzle voltage	2,000 V
Fragmentor	150 V
skimmer	65 V
OCT 1 RF Vpp	750 V
Collision Energy	25 V
Ion polarity	Negative

Table S3. LC-PDA conditions for ASE analysis

Instrument	Conditions		
**Column**	Capcell Pak C18 UG12, (4.6 mm x 250 mm, 5.0 μm)		
**Column temp.**	25℃		
**Mobile phase (Gradient)**	Time (min)	A<br>0.05% formic acid in water<br>(%)	B<br>Methanol<br>(%)

	0	95	5
	10	95	5
	15	85	15
	20	70	30
	30	60	40
	50	55	45
	52	40	60
	60	20	80
	65	95	5
**Detector**	Waters 996 Photodiode Array Detector (280 nm)		
**Flow rate**	0.8 mL/min		
**Run time**	65 min		
**Injection volume**	50 μL		
